# Network analysis of depression, anxiety, and stress symptoms in Brazilian nursing workers

**DOI:** 10.1590/0034-7167-2025-0675

**Published:** 2026-09-25

**Authors:** Lacir José Santin, Isabely Karoline da Silva Ribeiro, Samuel Andrade de Oliveira, Maynara Fernanda Carvalho Barreto, Ana Carolina Gomes Martins de Oliveira, Mayla Rodrigues Valadão Borges, Sérgio Joaquim Deodato Fernandes, Fernanda Ludmilla Rossi Rocha

**Affiliations:** I Universidade de São Paulo. Ribeirão Preto, São Paulo, Brazil; II Universidade Católica Portuguesa. Lisboa, Portugal

**Keywords:** Nursing, Depression, Anxiety, Stress, Psychological, Psychometrics., Enfermería, Depresión, Ansiedad, Estrés Psicológico, Psicometría.

## Abstract

**Objectives:**

to explore the network structure of depression, anxiety, and stress symptoms among Brazilian nursing workers.

**Methods:**

cross-sectional study using nonprobability sampling. Participants were 4,053 nursing workers. Symptoms of depression, anxiety, and stress were assessed using the Depression Anxiety Stress Scales-21. Data were analyzed using psychological network analysis, applying a partial correlation model with LASSO regularization method. Node centrality was assessed using strength, closeness, and betweenness indices.

**Results:**

the symptoms with the highest strength centrality were “I felt that I was using a lot of nervous energy,” “I was unable to become enthusiastic about anything,” and “I felt I was close to panic.” The symptom “I felt that I was using a lot of nervous energy” showed the highest closeness and betweenness indices.

**Conclusions:**

the identified central symptoms may play a structuring role in the activation of other symptoms and may help guide strategies to prevent and manage psychological distress among nursing workers.

## RESUMEN

**Objetivos:**

explorar la estructura de la red de síntomas de depresión, ansiedad y estrés en personal de enfermería brasileño.

**Métodos:**

estudio transversal con muestreo no probabilístico. Participaron 4.053 integrantes del personal de enfermería. Se utilizó la *Depression, Anxiety and Stress Scale* para evaluar síntomas. Los datos se analizaron mediante análisis de redes psicológicas, con modelo de correlaciones parciales y método de regularización LASSO. La centralidad de los nodos se evaluó mediante los índices de fuerza, cercanía e intermediación.

**Resultados:**

los síntomas con mayor fuerza de centralidad fueron “He sentido que estaba gastando una gran cantidad de energía”, “He sido incapaz de entusiasmarme por nada” y “He sentido que estaba al borde del pánico”. “He sentido que estaba gastando una gran cantidad de energía” también mostró los valores más altos de los índices de cercanía e intermediación.

**Conclusiones:**

los síntomas centrales identificados cumplen un papel clave en la activación de otros síntomas y pueden orientar estrategias de prevención y manejo del sufrimiento psicológico en enfermería.

## INTRODUCTION

The mental health of healthcare professionals has gained prominence in contemporary research, particularly given the high prevalence of symptoms of depression and anxiety. In Europe, a World Health Organization study conducted in 29 countries, involving more than 90,000 professionals (37,864 physicians and 52,307 nurses), found that one in three had depressive or anxiety symptoms and one in ten reported suicidal ideation^(1)^. These findings underscore that compromised mental health among these workers poses a concrete threat to the safety and functioning of health systems^(2)^.

Nursing accounts for approximately 59% of the global health workforce^(3)^. In Brazil, demographic data from the Annual Social Information Report (RAIS) indicate 12,932,405 nursing workers, including 4,183,526 (32.3%) nurses and 8,748,879 (67.7%) nursing technicians and nursing assistants between 2010 and 2021^(4)^. Nursing workers are continually exposed to adverse working conditions and multiple stressors, which significantly increase their vulnerability to mental disorders, particularly anxiety, depression, and stress^(3,5-7)^.

Recent evidence indicates that organizational, occupational, and individual factors contribute to mental illness in nursing and heighten vulnerability to psychological distress^(8)^. At the organizational level, work environments characterized by excessive workload, inadequate infrastructure, shortages of material and human resources, and strained interpersonal relationships are major sources of stress^(9,10)^. From an occupational perspective, long working hours, high technical demands, and accumulated responsibilities have been associated with increased symptoms of anxiety, depression, and stress^(11,12)^. Moreover, individual factors such as chronic comorbidities and low self-efficacy may exacerbate emotional symptoms, further increasing susceptibility to illness^(13)^.

Although these studies represent important advances, most available evidence focuses on the presence and severity of symptoms of anxiety, depression, and stress, rather than on how these symptoms relate to and influence one another^(14)^. Understanding symptom interactions is essential because mental illness is not merely a set of isolated manifestations; it functions as a dynamic system of interdependent elements that activate and reinforce one another and sustain complex emotional states^(14,15)^. This is precisely where network analysis emerges as a particularly relevant approach^(16)^. Over the past decades, this methodology has become established as an innovative tool in psychometric research by enabling the identification of patterns of statistical interconnection in multivariate psychological data^(16)^.

Unlike traditional models centered on latent constructs, network analysis conceptualizes symptoms as active units within a system, whose organization and reciprocal influence can be examined using centrality metrics such as strength, closeness, and betweenness^(15)^. This approach can help identify symptoms that occupy strategic positions, potentially contributing to the activation or intensification of other symptoms and thus playing a crucial role in mental illness among specific professional groups^(15,16)^.

Against this backdrop, the present study explores the symptom network structure of depression, anxiety, and stress among nursing workers in Brazil, providing a broader perspective on how these symptoms function in the workplace context.

## OBJECTIVES

To explore the network structure of depression, anxiety, and stress symptoms among Brazilian nursing workers.

## METHODS

### Ethical aspects

This study was approved by a Research Ethics Committee (CAAE 49679421.0.0000.5393; parecer 4.912.327). The study complied with Circular Letter No. 1/2021 issued by the National Research Ethics Commission, which guides research conducted in online settings, and with Resolution No. 738/2024 of the National Health Council, which addresses the use of databases for scientific research involving human participants.

### Study design, period, and setting

This cross-sectional study used nonprobability sampling and followed the Strengthening the reporting of observational studies in epidemiology (STROBE) guidelines. Data were collected from April to July 2022 in a fully online environment.

### Population or sample, inclusion and exclusion criteria

The sample comprised 4,053 nursing workers registered with the Federal Nursing Council (Cofen). Inclusion criteria were: being a nurse, nursing technician, or nursing assistant; having worked in nursing for at least one year; and being actively working during data collection. Participants who did not complete the instrument assessing symptoms of depression, anxiety, and stress were excluded.

### Study protocol

Nursing workers were invited to participate through an electronic message from Cofen. The message included a link to an online form built in Research Electronic Data Capture (REDCap) that contained the data collection instruments and the Informed Consent Form (ICF).

Two instruments were used for data collection. The first was designed to characterize participants, including individual information (age, sex, educational attainment, and marital status) and occupational information (occupational role, years of nursing experience, and weekly workload). The second was the psychometric instrument used to assess symptoms of depression, anxiety, and stress, the Depression Anxiety Stress Scales-21 (DASS-21)^(17)^. The DASS-21 was originally developed by Lovibond and Lovibond^(18)^ based on the Tripartite Model proposed by Clark and Watson^(19)^. The instrument comprises 21 items distributed across three factors: Depression (Items 3, 5, 10, 13, 16, 17, and 21), Anxiety (Items 2, 4, 7, 9, 15, 19, and 20), and Stress (Items 1, 6, 8, 11, 12, 14, and 18). Responses are recorded on a four-point Likert scale: 0 (Did not apply to me at all), 1 (Applied to me to some degree, or some of the time), 2 (Applied to me to a considerable degree or a good part of time), and 3 (Applied to me very much or most of the time). The DASS-21 has been previously validated among Brazilian nursing workers, demonstrating robust psychometric properties^(20)^.

### Data analysis and statistics

For data analysis, we used psychological network analysis, a graph-based statistical approach that allows visualization and interpretation of associations among multiple variables. These associations are represented in graphs, defined as sets of vertices (nodes) interconnected by edges (lines)^(21)^.

In network analyses of psychological data, nodes represent observed variables (psychological symptoms), whereas edges indicate the strength of connections between two nodes^(22,23)^. Blue edges represent positive associations, whereas red edges indicate negative associations. Edge thickness reflects the magnitude of these relationships. In the present study, nodes corresponded to DASS-21 items, and edges represented the interactions among them.

**Network estimation and regularization:** Relationships between items were examined using a partial correlation model^(24,25)^, with coefficients ranging from -1 to 1. For network regularization, the Least Absolute Shrinkage and Selection Operator (LASSO)^(26)^ method was used, which generates multiple networks based on the original data and reduces spurious or false-positive associations between variables. The most appropriate model was selected using the Extended Bayesian Information Criterion (EBIC)^(27)^. This fit measure uses the gamma hyperparameter (γ) to define the degree of penalization applied to spurious correlations, favoring parsimonious network structure^(28)^. In partial correlation network analyses, γ = 0.50 is considered the standard value^(22,27)^.

**Network description:** A node’s relevance in the network is determined by its centrality, an indicator of its structural and functional importance; higher strength centrality indicates greater importance within the network^(22)^. To quantify centrality, three indices were computed: (1) the strength index, defined as the sum of the weights of a node’s connections with all other nodes, which helps identify variables with stronger ties; (2) the betweenness index, which assesses how often a node lies on the shortest paths between pairs of nodes; and (3) the closeness index, defined as the inverse of the minimum average distance from a node to all others^(29)^. Indices were standardized as z scores (mean = 0; SD = 1), with values close to 1 indicating greater relevance in the network. For the graphical layout, the Fruchterman-Reingold algorithm was applied^(30)^ to optimize visualization of the nodes’ relative positions. However, closeness and betweenness indices may be unstable, especially in small samples^(16,22,31)^.

Estimation accuracy was assessed using nonparametric bootstrap with resampling with replacement, with a 95% confidence interval (95% CI). Network stability was examined using the case-dropping bootstrap and the correlation stability coefficient (CS); values below 0.25 indicate insufficient stability, values above 0.50 are considered acceptable, and values above 0.75 are considered the reference^(22)^.

All analyses were conducted in RStudio using the *pacman, qgraph, bootnet*, and *ggplot2* packages^(32)^.

## RESULTS

Table 1 presents the study participants’ characteristics.

**Table 1:** Participant characteristics (N = 4,053), Ribeirão Preto, São Paulo, Brazil, 2022

Variables	n	%
**Sex**		
Female	3,484	85.96
Male	543	13.40
No answer	26	0.64
**Age (years)**		
18-24	399	9.85
25-34	1,586	39.13
35-44	1,392	34.34
45-54	530	13.08
≥ 55	120	2.96
No answer	26	0.64
**Marital status**		
Never married	1,559	38.48
Married/common-law marriage	2036	50.23
Separated/divorced/widowed	449	11.07
No answer	09	0.22
**Professional category**		
Nurse	1,925	47.50
Nursing technician	1,902	46.93
Nursing assistant	212	5.23
No answer	14	0.34
**Years of nursing experience**		
1-5	1,754	43.29
6-10	1,029	25.39
11-15	621	15.32
16-20	322	7.94
21-25	161	3.97
26-30	78	1.92
> 30	40	0.99
No answer	48	1.18
**Multiple jobholding**		
Yes	1,374	33.90
No	2,644	65.24
No answer	35	0.86

The data in Table 1 show a predominance of female participants (85.96%), nurses (47.50%), and those who were married or in a common-law marriage (50.23%); most were aged 25-44 years (73.47%), had 1-5 years of nursing experience (43.29%), and did not hold more than one job (65.24%).

Regarding symptom network analysis, Figure 1 shows nodes based on DASS-21 items, forming three communities corresponding to the Depression, Anxiety, and Stress factors, shown in green, orange, and blue, respectively.

**Figure 1:**
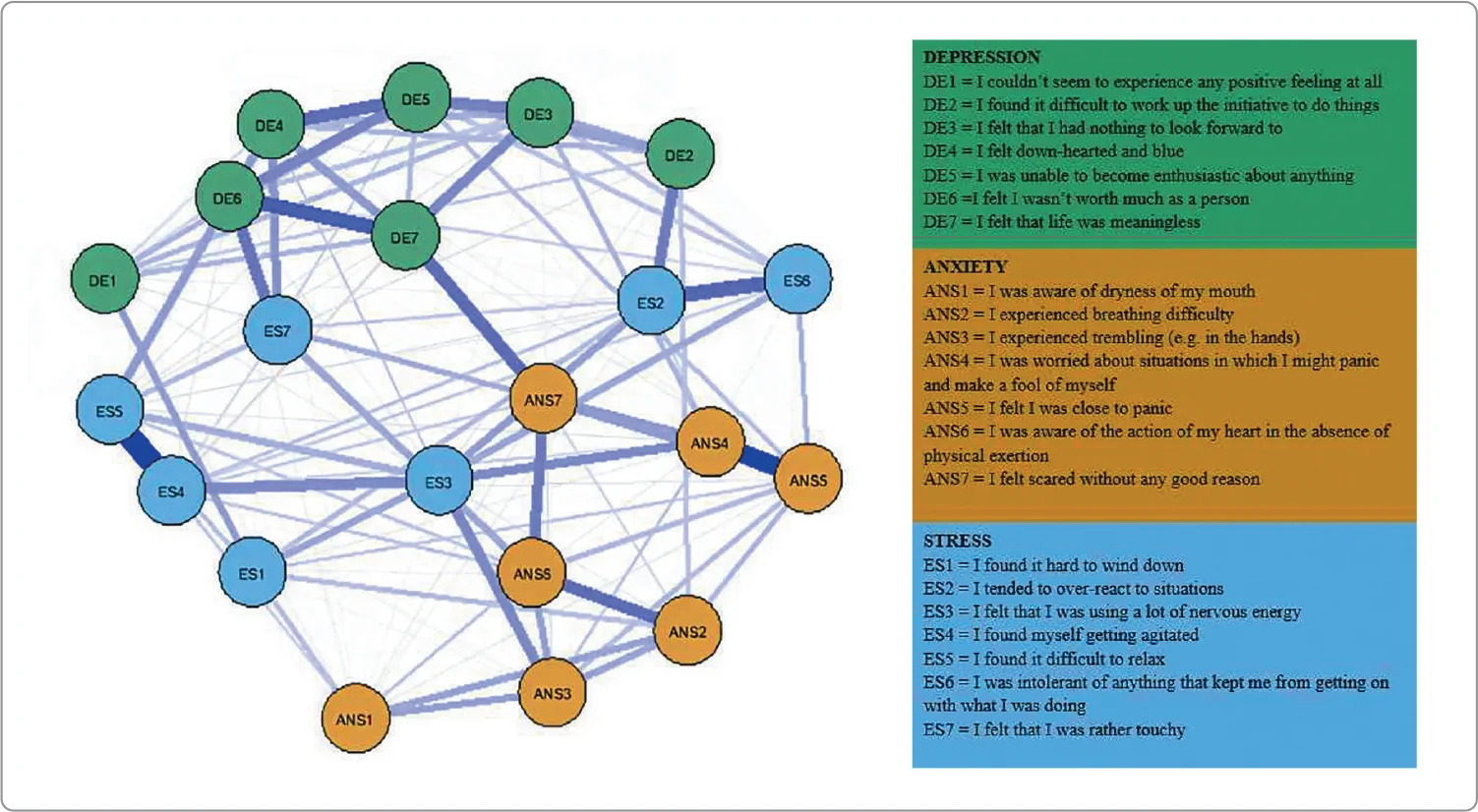
DASS-21 network analysis (N = 4,053), Ribeirão Preto, São Paulo, Brazil, 2022

The network showed interconnectedness among all nodes, with clearly defined clusters and the strongest edges occurring within clusters. Among depression symptoms, the strongest edges were between D6 (*I felt I wasn’t worth much as a person*) and D7 (*I felt that life was meaningless*), between D4 (*I felt down-hearted and blue*) and D5 (*I was unable to become enthusiastic about anything*), and between D3 (*I felt that I had nothing to look forward to*) and D5 (*I was unable to become enthusiastic about anything*).

Regarding anxiety symptoms, the strongest edges were between A4 (*I was worried about situations in which I might panic and make a fool of myself*) and A5 (*I felt I was close to panic*), between A2 (*I experienced breathing difficulty (e.g. excessively rapid breathing, breathlessness in the absence of physical exertion)*) and A6 (*I was aware of the action of my heart in the absence of physical exertion (e.g. sense of heart rate increase, heart missing a beat)*), and between A6 and A7 (*I felt scared without any good reason*).

Edge-weight analysis also indicated strong links between S4 (*I found myself getting agitated*) and S5 (*I found it difficult to relax*), as well as between A4 (*I was worried about situations in which I might panic and make a fool of myself*) and A5 (*I felt I was close to panic*). In addition, S3 (*I felt that I was using a lot of nervous energy*) was strongly connected to A3 (*I experienced trembling (e.g. in the hands)*) and A4 (*I was worried about situations in which I might panic and make a fool of myself*); S2 (*I tended to over-react to situations*) was strongly connected to D2 (*I found it difficult to work up the initiative to do things*); and S7 (*I felt that I was rather touchy*) was connected to D6 (*I felt I wasn’t worth much as a person*). Figure 2 shows the network’s centrality indices.

**Figure 2:**
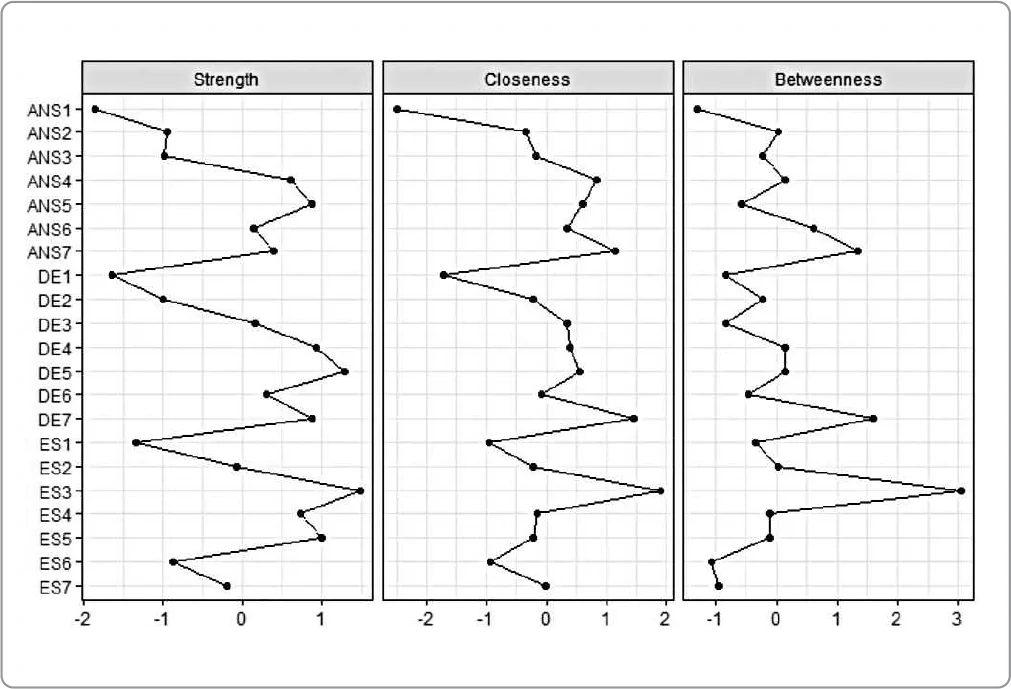
Network centrality indices (N = 4,053), Ribeirão Preto, São Paulo, Brazil, 2022

Strength centrality indicated that S3 (*I felt that I was using a lot of nervous energy*), D5 (*I was unable to become enthusiastic about anything*), and A5 (*I felt I was close to panic*) were the most central symptoms in the network. S3 also had the highest closeness and betweenness, indicating that this symptom is particularly relevant to how nodes interrelate and to the network’s overall connectivity.

Figure 3 shows the accuracy of network estimation for the symptom network of depression, anxiety, and stress among participants, indicating that across successive resampling iterations, the strength centrality index showed the greatest stability, whereas the other indices declined slightly-especially after 50% of cases were dropped.

**Figure 3:**
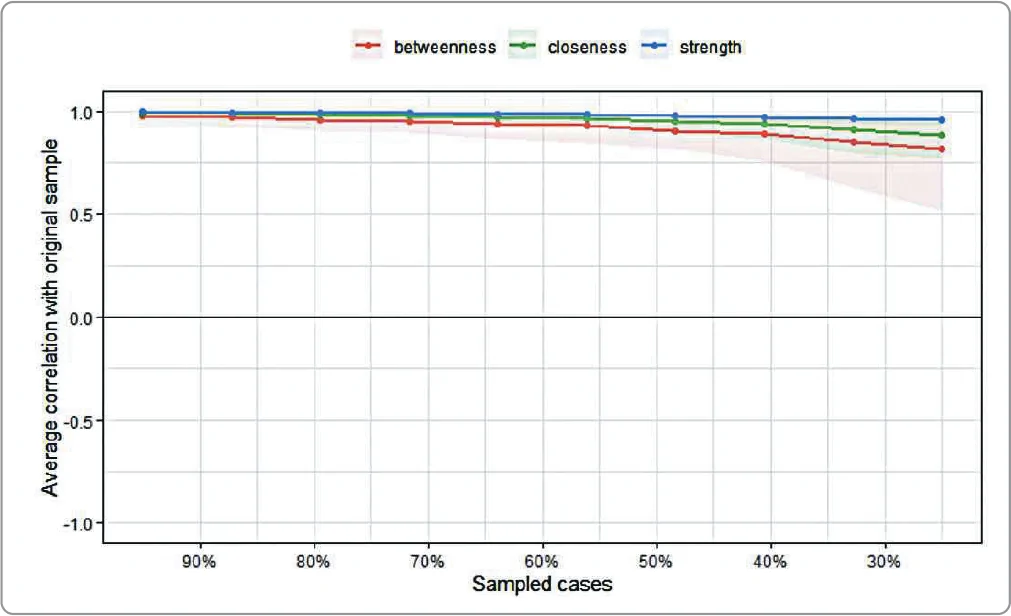
Accuracy of network estimation (N = 4,053), Ribeirão Preto, São Paulo, Brazil, 2022

Regarding network stability, the CS coefficient for strength centrality was 0.75; the CS values for closeness and betweenness were also acceptable (0.75 and 0.59, respectively).

## DISCUSSION

This study aimed to explore the network structure of depression, anxiety, and stress symptoms among Brazilian nursing workers, offering a broader perspective on how these symptoms interrelate and mutually influence work-related mental health problems in this population.

Participants’ individual characteristics reflected the current profile of the nursing workforce in Brazil and across the Americas, as described in the report “Nursing in the Region of the Americas 2025”^(33,34)^, which highlights the predominance of women and the relatively young workforce.

In the network analysis, nodes clustered into three clearly defined communities corresponding to the DASS-21 factors (Depression, Anxiety, and Stress). According to the Tripartite model^(19)^-the theoretical premise underlying the development of the DASS-21-depression and anxiety can be characterized by specific symptoms: depression by anhedonia (reduced capacity to experience pleasure, loss of interest, and low energy), sadness, dysphoria (a persistent state of unhappiness and dissatisfaction), hopelessness, devaluation of life, and self-deprecation; and anxiety by physiological hyperarousal (palpitations, trembling, sweating, and shortness of breath), muscle tension, nervousness, a persistent perception of threat or danger, and emotional instability. Some symptoms, however, are nonspecific or shared by both conditions, such as sadness, fear, irritability, worry, poor concentration, insomnia, fatigue, guilt, and low self-esteem. Thus, the Tripartite model comprises two specific factors (depression and anxiety) and a third factor composed of nonspecific symptoms (the distress factor) that, although distinct, is closely related to the other factors because of conceptual proximity and clinical overlap. In developing the DASS-21, symptoms of a mixed or shared nature were allocated to the “Stress” factor.

This framework was supported by strong within-cluster edges. The strong edge between D6 (*I felt I wasn’t worth much as a person*) and D7 (*I felt that life was meaningless*) reflected anhedonia and diminished personal motivation, which are central symptoms of depression; the strong edge between A4 (*I was worried about situations in which I might panic and make a fool of myself*) and A5 (*I felt I was close to panic*) highlighted physiological hyperarousal as a hallmark of anxiety; and the strong edge between S4 (*I found myself getting agitated*) and S5 (*I found it difficult to relax*) underscored the core of tension and irritability characteristic of stress.

The edge between S3 (*I felt that I was using a lot of nervous energy*) and anxiety symptoms such as A3 (*I experienced trembling (e.g. in the hands)*) and A4 (*I was worried about situations in which I might panic and make a fool of myself*) also points to overlap in nonspecific or shared symptoms between anxiety and stress states, including nervousness, worry, and hyperarousal. This pattern further suggests that the stress response involves not only emotional components but also physiological manifestations. When emotional and physical responses are activated simultaneously, they tend to reinforce one another, amplifying psychological distress^(18,19)^.

Given their structural role in the network, the nodes with the highest strength centrality were the depression symptom D5 (*I was unable to become enthusiastic about anything*), the anxiety symptom A5 (*I felt I was close to panic*), and the stress symptom S3 (*I felt that I was using a lot of nervous energy*). These symptoms again emerged as key nodes in the network of physical and psychological manifestations associated with participants’ psychological distress.

Additionally, S3 (*I felt that I was using a lot of nervous energy*) showed the highest strength, closeness, and betweenness, suggesting that this symptom occupied a strategic position in the network by connecting different symptom clusters. This finding indicates that, beyond having stronger ties to other nodes, it tended to be closer to the rest of the network and to lie more often on the shortest paths between nodes, supporting its role as a key bridge across clusters. In other words, S3 (*I felt that I was using a lot of nervous energy*) was one of the most prominent symptoms among participants and was broadly connected across the network of mental health symptoms among nursing workers. These results are consistent with findings from a network analysis conducted with 11,647 participants from multiple countries^(14)^ and highlight the close conceptual relationship between stress and states of depression and anxiety^(18,19)^.

The relevance of S3 (*I felt that I was using a lot of nervous energy*) in the symptom network also underscores the importance of stress in work-related mental health problems among professionals who are continually exposed to high work demands, such as health workers, particularly nursing workers. These professionals face daily contexts marked by work overload^(35,36)^, resource shortages, and high psychoemotional demands related to caregiving^(8,10)^-psychosocial factors widely associated with physical and mental health problems in this workforce.

Recent studies using the DASS-21 among nursing workers have identified individual and occupational factors associated with higher symptom levels. At the individual level, fewer years of professional experience^(37)^, the presence of comorbidities^(13)^, and alcohol consumption^(10)^ have been associated with higher levels of depression, anxiety, and stress. In the occupational context, long working hours^(11)^, shortages of human and material resources^(9)^, and job dissatisfaction^(10)^ have been identified as additional risk factors. This body of evidence reinforces that the symptom pattern observed reflects the interaction between individual vulnerabilities and working conditions, highlighting the need for care strategies that integrate personal and organizational dimensions.

International studies on depression, anxiety, and stress among nurses conducted during the COVID-19 pandemic reported exacerbation of symptoms during that period^(7,38,39)^. This context was marked by intensified emotional distress among nursing workers, who faced a prolonged fear of contamination^(38)^, a marked increase in workload^(40)^, and direct exposure to high patient mortality^(41)^. Notably, the effects of the pandemic persisted even after the critical phase of the health crisis, according to World Health Organization and United Nations reports^(42,43)^, indicating the prolonged impact of this event on professionals’ physical and mental health.

In light of these findings, the results of this study reinforce the Tripartite Model as a theoretical framework for understanding mental health problems related to stress, anxiety, and depression among nursing workers. By highlighting central symptoms that play a strategic role in the network, the analysis clarifies how these elements are organized and interact, extending the model’s applicability to the context of healthcare work.

### Study limitations

Because instruments specifically designed to assess the organizational context were not used, the network model did not incorporate relevant external variables such as working conditions, resource availability, and organizational support. As a result, although the network captured the dynamic relationships among symptoms of depression, anxiety, and stress, its interpretation does not allow a precise assessment of organizational conditions that may be related to work-related mental health problems among nursing workers. Moreover, this study design precludes causal inference among the variables examined.

### Contributions to the field of nursing

Network analysis of the DASS-21 among nursing workers makes an important contribution by showing how symptoms of depression, anxiety, and stress are interconnected and may mutually reinforce one another. This approach helped identify the symptoms most central to the network and to overall connectivity, suggesting a symptom pattern specific to this occupational group. These findings may inform workers’ self-care strategies and support health service managers in planning more targeted actions focused on the symptoms that most affect team well-being. In addition, the study contributes to nursing by expanding the evidence base supported by robust methods and offering an additional lens for understanding work-related mental health problems.

## CONCLUSIONS

The results of this study indicate that work-related mental health problems among nursing workers are structured around central symptoms that occupy key positions in the network, with broad connections across manifestations of depression, anxiety, and stress. Identifying these central symptoms highlights structural patterns that help clarify how emotional distress is articulated within this workforce, pointing to vulnerabilities that, when unrecognized, may amplify the psychosocial harms of work.

These findings strengthen the potential of the DASS-21 as a symptom screening tool and provide solid guidance for managers, researchers, and policymakers in developing strategies to prevent and manage work-related psychosocial risks. By indicating which symptoms are most central in the network, the analysis can help guide more targeted and effective interventions. Overall, the results underscore the need for institutional policies that promote psychologically safe work environments, including continuous monitoring and psychosocial support. Working conditions also require reorganization to help reduce distress and promote nursing workers’ mental health.

## Data Availability

The research data are available only upon request.
