# Supplementary material for: Network analysis of depression, anxiety, and stress symptoms in Brazilian nursing workers
Source: Rev Bras Enferm. 2026 Sep 25;79:e20250675. doi: 10.1590/0034-7167-2025-0675 (PMC13619155; doi:10.1590/0034-7167-2025-0675)
Supplement: Translation

# Translation: Análise de rede de sintomas de depressão, ansiedade e estresse entre trabalhadores de enfermagem brasileiros

**DOI:** 10.1590/0034-7167-2025-0675pt

**Authors:** Lacir José Santin, Isabely Karoline da Silva Ribeiro, Samuel Andrade de Oliveira, Maynara Fernanda Carvalho Barreto, Ana Carolina Gomes Martins de Oliveira, Mayla Rodrigues Valadão Borges, Sérgio Joaquim Deodato Fernandes, Fernanda Ludmilla Rossi Rocha

**Affiliations:** I Universidade de São Paulo. Ribeirão Preto, São Paulo, Brasil; II Universidade Católica Portuguesa. Lisboa, Portugal

**Keywords:** Enfermagem, Depressão, Ansiedade, Estresse Psicológico, Psicometria.

## Abstract

**Objetivos:**

explorar a estrutura da rede de sintomas de depressão, ansiedade e estresse entre trabalhadores de enfermagem brasileiros.

**Métodos:**

estudo transversal com amostragem não probabilística. Participaram 4.053 profissionais de enfermagem. Utilizou-se a *Depression, Anxiety and Stress Scale* para avaliar sintomas de depressão, ansiedade e estresse. Analisaram-se os dados mediante a técnica da análise de rede psicológica, utilizando-se o modelo de correlação parcial e o método de regressão Lasso. A centralidade dos nós foi avaliada pelos índices de força, proximidade e intermediação.

**Resultados:**

os sintomas com maior força de centralidade foram: “Estar sempre nervoso”, “Não conseguir se entusiasmar” e “Sentir que iria entrar em pânico”. O sintoma “Estar sempre nervoso” também apresentou os maiores índices de proximidade e de intermediação.

**Conclusões:**

os sintomas centrais identificados exercem papel estruturante na ativação dos demais e podem orientar estratégias de prevenção e manejo do sofrimento psíquico na enfermagem.

## INTRODUÇÃO

A saúde mental de profissionais de saúde tem adquirido centralidade nas pesquisas contemporâneas, especialmente diante da elevada prevalência de sintomas de depressão e ansiedade. Na Europa, um estudo conduzido pela Organização Mundial da Saúde em 29 países, envolvendo mais de 90 mil profissionais (37.864 médicos e 52.307 enfermeiros), identificou que um em cada três apresentou sintomas depressivos ou ansiosos, e um em cada dez relatou ideação suicida^(1)^. Esses resultados reforçam que o comprometimento da saúde mental desses trabalhadores representa uma ameaça concreta à segurança e ao funcionamento dos sistemas de saúde^(2)^.

A enfermagem corresponde a aproximadamente 59% da força de trabalho em saúde no mundo^(3)^. No Brasil, dados demográficos provenientes da Relação Anual de Informações Sociais (RAIS) apontam a existência de 12.932.405 profissionais de enfermagem, distribuídos entre 4.183.526 (32,3%) enfermeiros e 8.748.879 (67,7%) técnicos e auxiliares entre 2010 e 2021^(4)^. Toda a categoria está continuamente exposta a condições laborais adversas e a múltiplos fatores estressores, os quais aumentam significativamente sua vulnerabilidade ao desenvolvimento de transtornos mentais, especialmente ansiedade, depressão e estresse^(3,5-7)^.

Evidências recentes indicam que um conjunto de fatores organizacionais, ocupacionais e individuais gera o adoecimento mental na enfermagem e aumenta sua vulnerabilidade ao sofrimento psíquico^(8)^. No âmbito organizacional, ambientes marcados por sobrecarga assistencial, infraestrutura inadequada, escassez de recursos materiais e humanos e relações interpessoais tensionadas configuram importantes fontes de estresse^(9,10)^. Do ponto de vista ocupacional, longas jornadas de trabalho, alta exigência técnica e acúmulo de responsabilidades têm sido associados ao aumento de sintomas de ansiedade, depressão e estresse^(11,12)^. Ademais, fatores individuais, como presença de comorbidades crônicas e baixa autoeficácia, também contribuem para o agravamento da sintomatologia emocional, ampliando a suscetibilidade ao adoecimento^(13)^.

Embora esses estudos ofereçam avanços relevantes, a maior parte das evidências disponíveis focaliza a presença e a intensidade dos sintomas de ansiedade, depressão e estresse, mas não a forma como eles se relacionam e se influenciam mutuamente^(14)^. Na atualidade, compreender a interação entre sintomas é fundamental, pois o adoecimento mental não opera enquanto um conjunto isolado de manifestações, mas na qualidade de um sistema dinâmico de elementos interdependentes que se ativam e se retroalimentam, bem como sustentam estados emocionais complexos^(14,15)^. É precisamente nesse ponto que a análise de rede se apresenta como uma abordagem particularmente relevante^(16)^. Nas últimas décadas, essa metodologia consolidou-se como uma ferramenta inovadora na pesquisa psicométrica ao permitir a identificação de padrões de interconexão estatística em dados psicológicos multivariados^(16)^.

Diferentemente de modelos tradicionais centrados em construtos latentes, a análise de rede concebe os sintomas como unidades ativas de um sistema, cuja organização e influência recíproca podem ser examinadas por meio de métricas de centralidade, força, proximidade e intermediação^(15)^. Assim, essa abordagem possibilita compreender quais sintomas ocupam posições estratégicas, desencadeiam ou amplificam outros sintomas e, portanto, desempenham papel crucial no adoecimento mental de determinados grupos profissionais^(15,16)^.

Diante desse panorama, o presente estudo explora a estrutura da rede de sintomas de depressão, ansiedade e estresse em trabalhadores de enfermagem brasileiros, fornecendo uma perspectiva ampliada sobre o funcionamento desses sintomas no contexto laboral.

## OBJETIVOS

Explorar a estrutura da rede de sintomas de depressão, ansiedade e estresse entre trabalhadores de enfermagem brasileiros.

## MÉTODOS

### Aspectos éticos

Este estudo foi aprovado por um Comitê de Ética em Pesquisa (CAAE 49679421.0.0000.5393; parecer 4.912.327). Foram seguidas todas as diretrizes da Carta Circular 1/2021 do Conselho Nacional de Ética em Pesquisa, referente às orientações para procedimentos em pesquisas em ambiente virtual, e da Resolução 738/2024 do Conselho Nacional de Saúde, que dispõe sobre o uso de bancos de dados com finalidade de pesquisa científica envolvendo seres humanos.

### Desenho, período e local do estudo

Trata-se de estudo transversal, com amostragem não probabilística. Seguiu as recomendações do *Strengthening the reporting of observational studies in epidemiology* (STROBE). A coleta de dados foi realizada entre abril e julho de 2022, em ambiente totalmente virtual.

### População ou amostra, critérios de inclusão e exclusão

A amostra foi composta por 4.053 profissionais de enfermagem cadastrados no Conselho Federal de Enfermagem (Cofen). Os critérios de inclusão foram: ser enfermeiro, técnico ou auxiliar de enfermagem; trabalhar na área da enfermagem há pelo menos um ano; e estar atuando na profissão no período da coleta de dados. Foram excluídos da amostra os participantes que não responderam completamente o instrumento de avaliação de sintomas de depressão, ansiedade e estresse.

### Protocolo do estudo

Os profissionais de enfermagem foram convidados a participar do estudo por meio de mensagem eletrônica enviada pelo Conselho Federal de Enfermagem (COFEN). Essa mensagem incluía um link de acesso a um formulário eletrônico elaborado na plataforma *Research Electronic Data Capture* (REDCap), composto pelos instrumentos de coleta de dados e pelo Termo de Consentimento Livre e Esclarecido (TCLE).

Foram disponibilizados dois instrumentos para a coleta de dados. O primeiro destinava-se à caracterização dos participantes, abrangendo informações individuais (idade, sexo, escolaridade e estado civil) e ocupacionais (função exercida, tempo de trabalho na enfermagem e carga horária semanal). O segundo era o instrumento psicométrico de avaliação de sintomas de depressão, ansiedade e estresse, a *Depression, Anxiety and Stress Scale* - DASS-21^(17)^. A DASS-21 foi originalmente desenvolvida por *Lovibond* e *Lovibond* ^(18)^, com base no Modelo Tripartido proposto por Clark e Watson^(19)^. O instrumento é composto por 21 itens distribuídos em três fatores: Depressão (Itens 3, 5, 10, 13, 16, 17 e 21), Ansiedade (Itens 2, 4, 7, 9, 15, 19 e 20) e Estresse (Itens 1, 6, 8, 11, 12, 14 e 18). As respostas são registradas em uma escala Likert de quatro pontos: 0 - não se aplicou de maneira alguma; 1 - aplicou-se em algum grau ou por pouco tempo; 2 - aplicou-se em grau considerável ou por boa parte do tempo; 3 - aplicou-se muito ou na maioria do tempo. Destaca-se que a DASS-21 foi previamente validada entre trabalhadores de enfermagem brasileiros, apresentando propriedades psicométricas robustas^(20)^.

### Análise dos resultados e estatística

Para a análise dos dados, empregou-se a análise de rede psicológica, uma técnica estatística de caráter gráfico que possibilita visualizar e interpretar as associações entre múltiplas variáveis. Essas associações são visualizadas por meio de “grafos”, os quais são definidos como conjuntos de vértices (nós) interconectados por arestas (linhas)^(21)^.

Nas análises de rede aplicadas a dados psicológicos, os nós correspondem às variáveis observáveis (sintomas psicológicos), enquanto as arestas indicam a intensidade das conexões entre duas ou mais variáveis^(22,23)^. As arestas em azul representam associações positivas, ao passo que as vermelhas sinalizam associações negativas. Já a espessura ou densidade das arestas expressa a magnitude dessas relações. No presente estudo, os nós correspondem aos itens da DASS-21, e as arestas descrevem as interações estabelecidas entre eles.

**Estimação e regularização da rede:** as relações entre os itens foram investigadas por meio do modelo de correlação parcial^(24,25)^, cujos coeficientes variam entre -1 e 1. Para a regularização da rede, utilizou-se o método *Least Absolute Shrinkage and Selection Operator* (LASSO)^(26)^, que gera múltiplas redes com base nos dados originais e reduz a ocorrência de associações espúrias ou falso positivas entre variáveis. A seleção do modelo mais adequado foi realizada apoiando-se no critério *Extended Bayesian Information Criteria* (EBIC)^(27)^. Essa medida de ajuste utiliza o hiperparâmetro gama (γ) para definir o grau de penalização aplicado às correlações espúrias, favorecendo uma estrutura de rede parcimoniosa^(28)^. Nas análises de redes de correlações parciais, o valor γ = 0,50 é considerado padrão^(22,27)^.

**Descrição da rede:** a relevância de um nó na rede é determinada por sua centralidade, indicador que reflete sua importância estrutural e funcional; quanto maior a força de centralidade, maior a significância do nó^(22)^. Para mensurar a centralidade, foram calculados três índices: (1) índice de força (*strength*), que corresponde à soma dos pesos das conexões de um nó com os demais, permitindo identificar variáveis com vínculos mais robustos; (2) índice de intermediação (*betweenness*), que avalia a frequência com que um nó aparece nos caminhos mais curtos entre pares de nós; e (3) índice de proximidade (*closeness*), definido como o inverso da distância média mínima de um nó em relação a todos os outros^(29)^. Os índices foram padronizados em escores z (média = 0; desvio-padrão = 1), de modo que valores próximos a 1 indicam maior relevância na rede. Para a disposição gráfica, foi aplicado o algoritmo de Fruchterman-Reingold^(30)^, que otimiza a visualização das posições relativas entre os nós. Contudo, ressalta-se que os índices de proximidade e intermediação podem apresentar instabilidade, especialmente em amostras pequenas^(16,22,31)^.

A precisão das estimativas foi avaliada por meio do método *bootstrap* não paramétrico de queda de caso, com intervalo de confiança de 95% (IC 95%), que realiza múltiplas reamostragens com reposição da amostra original. A estabilidade da rede foi examinada pelo coeficiente de estabilidade de correlação (CS), em que valores abaixo de 0,25 indicam estabilidade insuficiente, valores acima de 0,50 são considerados aceitáveis, e valores superiores a 0,75 são considerados a referência^(22)^.

Todas as análises foram conduzidas no software RStudio, utilizando os pacotes *pacman, qgraph, bootnet* e *ggplot2* ^(32)^.

## RESULTADOS

A Tabela 1 apresenta a caracterização dos participantes do estudo.

**Tabela 1:** Caracterização dos participantes (N = 4.053), Ribeirão Preto, São Paulo, Brasil, 2022

Variáveis	n	%
**Sexo**		
Feminino	3.484	85,96
Masculino	543	13,40
Não responderam	26	0,64
**Idade (anos)**		
18 a 24	399	9,85
25 a 34	1.586	39,13
35 a 44	1.392	34,34
45 a 54	530	13,08
≥ 55	120	2,96
Não responderam	26	0,64
**Estado civil**		
Solteiro(a)	1.559	38,48
Casado/união estável	2036	50,23
Separado/divorciado/viúvo	449	11,07
Não responderam	09	0,22
**Categoria profissional**		
Enfermeiro(a)	1.925	47,50
Técnico de enfermagem	1.902	46,93
Auxiliar de enfermagem	212	5,23
Não responderam	14	0,34
**Tempo de trabalho na enfermagem (anos)**		
1 a 5	1.754	43,29
6 a 10	1.029	25,39
11 a 15	621	15,32
16 a 20	322	7,94
21 a 25	161	3,97
26 a 30	78	1,92
> 30	40	0,99
Não responderam	48	1,18
**Duplo vínculo**		
Sim	1.374	33,90
Não	2.644	65,24
Não responderam	35	0,86

Os dados da Tabela 1 mostram a predominância de mulheres (85,96%); profissionais enfermeiros (47,50%); casados ou em união estável (50,23%); com idade entre 25 e 44 anos (73,47%); experiência na enfermagem entre 1 e 5 anos (43,29%); sem duplo vínculo empregatício (65,24%).

Quanto à análise de rede dos sintomas, a Figura 1 mostra os nós organizados com base nos itens da DASS-21, formando três comunidades ou agrupamentos referentes aos fatores Depressão, Ansiedade e Estresse, identificados pelas cores verde, laranja e azul, respectivamente.

**Figura 1:** Análise de rede da DASS-21 (N = 4.053), Ribeirão Preto, São Paulo, Brasil, 2022

Observou-se a interconectividade entre todos os nós, a definição clara dos agrupamentos e as conexões mais fortes entre nós pertencentes ao mesmo agrupamento da rede. Entre os sintomas de depressão, as conexões mais fortes ocorreram entre os nós DE6 (*Senti que não tinha valor como pessoa*) e DE7 (*Senti que a vida não tinha sentido*); entre DE4 (*Senti-me depressivo[a] e sem ânimo*) e DE5 (*Não consegui me entusiasmar com nada*); e entre DE3 (*Senti que não tinha nada a desejar*) e DE5.

Sobre os sintomas de ansiedade, as conexões mais fortes foram atestadas entre os nós ANS4 (*Preocupei-me com situações em que eu pudesse entrar em pânico e parecesse ridículo[a]*) e ANS5 (*Senti que ia entrar em pânico*); entre ANS2 (*Tive dificuldade em respirar em alguns momentos [p.ex., respiração ofegante, falta de ar, sem ter feito nenhum esforço físico]*) e ANS6 (*Sabia que meu coração estava alterado mesmo não tendo feito nenhum esforço físico [p.ex., aumento da frequência cardíaca, disritmia cardíaca]*); e entre ANS6 e ANS7 (*Senti medo sem motivo algum*).

A análise das conexões entre os nós mostrou que as conexões mais fortes ocorreram entre ES4 (*Senti-me agitado*) e ES5 (*Achei difícil relaxar*); e entre ANS4 (*Preocupei-me com situações em que eu pudesse entrar em pânico e parecesse ridículo[a]*) e ANS5 (*Senti que ia entrar em pânico)*. Além disso, o sintoma de estresse ES3 (*Senti que estava sempre nervoso*) teve conexões significativas com os sintomas de ansiedade ANS3 (Senti tremores [p.ex., nas mãos]) e ANS4 (*Preocupei-me com situações em que eu pudesse entrar em pânico e parecesse ridículo[a]*); o sintoma ES2 (*Tive a tendência de reagir de forma exagerada às situações*) mostrou conexões importantes com o sintoma de depressão DE2 (*Achei difícil ter iniciativa para fazer as coisas)*; e o sintoma ES7 (Senti que estava um pouco emotivo/sensível demais) apresentou conexão com o sintoma DE6 (*Senti que não tinha valor como pessoa*). A Figura 2 mostra os índices de centralidade da rede.

**Figura 2:** Índices de centralidade dos nós (N = 4.053), Ribeirão Preto, São Paulo, Brasil, 2022

A análise da força de centralidade (*strength*) revelou que os nós ES3 (*Senti que estava sempre nervoso)*, DE5 (*Não consegui me entusiasmar com nada*) e ANS5 (*Senti que ia entrar em pânico)* foram os sintomas mais importantes da rede. Ressalta-se que o nó ES3 obteve também os maiores índices de proximidade (*closeness)* e de intermediação (*betweenness)*, indicando que esse sintoma é também o mais relevante para o inter-relacionamento entre todos os nós e para a conectividade da rede como um todo.

A Figura 3 mostra a precisão da estimação da rede de sintomas de depressão, ansiedade e estresse entre os participantes, indicando que, após sucessivas reamostragens, o índice de força de centralidade (*strength*) foi o mais estável, enquanto os demais tiveram ligeira queda, principalmente após a exclusão de 50% dos casos.

**Figura 3:** Precisão da estimação da rede (N = 4.053), Ribeirão Preto, São Paulo, Brasil, 2022

Quanto à estabilidade da rede, o coeficiente de estabilidade (CS) relacionado à força de centralidade foi de 0,75; os coeficientes de proximidade (*closeness*) e de intermediação (*betweenness*) também atingiram valores aceitáveis (0,75 e 0,59, respectivamente).

## DISCUSSÃO

O presente estudo teve como objetivo explorar a estrutura da rede dos sintomas de depressão, ansiedade e estresse entre trabalhadores de enfermagem brasileiros, buscando uma perspectiva ampliada para a compreensão da inter-relação e da influência mútua desses sintomas no processo de adoecimento mental relacionado ao trabalho dessa população.

As características individuais dos participantes refletiram o perfil atual da força de trabalho em enfermagem no Brasil e nas Américas, conforme descrito no relatório “Enfermagem na Região das Américas 2025”^(33,34)^, que aponta predominância feminina e relativa juventude profissional.

Em relação à análise de rede, esta revelou agrupamentos de nós que refletiram três comunidades bem definidas, representando os fatores da DASS-21 (Depressão, Ansiedade e Estresse). Com base no Modelo Tripartido^(19)^, premissa teórica que subsidiou o desenvolvimento da DASS-21, a depressão e a ansiedade podem ser caracterizadas por sintomas específicos, tais como: no caso da depressão, anedonia (capacidade reduzida de sentir prazer, perda de interesse e baixa energia), tristeza, disforia (estado persistente de infelicidade e insatisfação), desesperança, desvalorização da vida e autodepreciação; e, no caso da ansiedade, hiperexcitação fisiológica (palpitações, tremores, sudorese e falta de ar), tensão muscular, nervosismo, percepção constante de ameaça ou perigo e instabilidade. Entretanto, alguns sintomas são inespecíficos ou podem ser compartilhados entre as duas condições, como sentimentos de tristeza, medo, irritabilidade, preocupação, baixa concentração, insônia, fadiga, culpa e baixa autoestima. Assim, o Modelo Tripartido é composto por dois fatores específicos (depressão e ansiedade) e por um fator composto por sintomas inespecíficos, conhecido como *distress factor*, o qual, apesar de ser considerado distinto dos outros fatores, está estreitamente relacionado a eles, dada a proximidade conceitual e a sobreposição clínica existente entre os sintomas. Na elaboração da DASS-21, sintomas com natureza mista ou compartilhada foram alocados no fator “Estresse”.

Esses pressupostos teóricos foram confirmados por meio da observação de fortes conexões entre nós do mesmo agrupamento da rede. Assim, a forte conexão entre DE6 (*Senti que não tinha valor como pessoa*) e DE7 (*Senti que a vida não tinha sentido*) refletiu anedonia e perda de motivação pessoal, que são sintomas centrais da depressão; a forte conexão entre ANS4 (*Preocupei-me com situações em que eu pudesse entrar em pânico e parecesse ridículo[a]*) e ANS5 *(Senti que ia entrar em pânico)* comprovou a hiperativação fisiológica como marca da ansiedade; e a forte conexão entre ES4 (*Senti-me agitado*) e ES5 (*Achei difícil relaxar)* evidenciou o núcleo de tensão e irritabilidade característico do estresse.

Já a conexão entre ES3 *(Senti que estava sempre nervoso)* e sintomas ansiosos, como ANS3 (*Senti tremores [ex. nas mãos]*) e ANS4 (*Preocupei-me com situações em que eu pudesse entrar em pânico e parecesse ridículo[a]*), comprovou a sobreposição de sintomas inespecíficos ou compartilhados entre estados de ansiedade e de estresse, como nervosismo, preocupação e hiperexcitação. Essa relação também sugere que a resposta ao estresse envolve não apenas componentes emocionais, mas também manifestações fisiológicas. Quando respostas emocionais e físicas são ativadas simultaneamente, elas tendem a se potencializar, ampliando a experiência de sofrimento mental^(18,19)^.

Considerando o papel estrutural desses sintomas na rede, os nós que demonstraram os maiores índices de força de centralidade na rede foram o sintoma de depressão DE5 (*Não consegui me entusiasmar com nada*); de ansiedade ANS5 (*Senti que ia entrar em pânico*); e de estresse ES3 (*Senti que estava sempre nervoso*). Novamente, esses sintomas apareceram como determinantes da rede de manifestações físicas e psicológicas associadas ao sofrimento mental dos participantes.

Ainda, a análise evidenciou que o nó ES3 (*Senti que estava sempre nervoso*) obteve os maiores índices de força, proximidade e intermediação, indicando que esse sintoma ocupa uma posição estratégica na rede ao conectar diferentes grupos de sintomas. Isso significa que, além de manter relações mais intensas com os demais nós, exerce influência expressiva sobre o fluxo das interações, já que se configura como o nó com menores distâncias em relação aos outros e atua enquanto intermediário essencial na articulação entre diferentes conjuntos de sintomas. Em outras palavras, “sentir-se sempre nervoso” foi o sentimento mais forte entre os participantes e aquele que mais influenciou a ocorrência de todos os outros sintomas de adoecimento mental dos trabalhadores de enfermagem. Esses resultados corroboram os achados de análise de rede conduzida com 11.647 participantes de diversos países^(14)^ e comprovam a estreita relação conceitual entre o estresse e os estados de depressão e ansiedade^(18,19)^.

A relevância do nó ES3 (*Senti que estava sempre nervoso)* na rede de sintomas também demonstra a importância do estresse como fator determinante do adoecimento de profissionais continuamente expostos a altas demandas de trabalho, como os trabalhadores de saúde, especialmente de enfermagem. Esses profissionais enfrentam diariamente contextos marcados pela sobrecarga de trabalho^(35,36)^, escassez de recursos e elevadas demandas psicoemocionais relacionadas ao cuidado^(8,10)^ - fatores psicossociais amplamente associados ao adoecimento físico e mental desses trabalhadores.

Estudos recentes que utilizaram a DASS-21 em trabalhadores de enfermagem mostraram os principais fatores individuais e ocupacionais que contribuem para o agravamento da sintomatologia. No âmbito individual, menor tempo de experiência profissional^(37)^, presença de comorbidades^(13)^ e consumo de álcool^(10)^ estão associados a níveis mais elevados de depressão, ansiedade e estresse. No contexto ocupacional, extensas jornadas de trabalho^(11)^, escassez de recursos humanos e materiais^(9)^ e insatisfação com o trabalho^(10)^ foram identificados como fatores de risco adicionais. Esse conjunto de evidências reforça que a sintomatologia observada resulta da interação entre vulnerabilidades individuais e condições de trabalho, destacando a necessidade de estratégias de cuidado que integrem dimensões pessoais e organizacionais.

Pesquisas internacionais sobre a ocorrência de depressão, ansiedade e estresse entre enfermeiros realizadas durante a pandemia da covid-19 demonstraram a exacerbação de sintomas nesse período^(7,38,39)^. Trata-se de um contexto marcado pela intensificação do sofrimento emocional de trabalhadores da enfermagem, os quais foram expostos a um longo período de medo de contaminação^(38)^, aumento expressivo da carga de trabalho^(40)^ e vivência direta de alta mortalidade entre pacientes^(41)^. Ressalta-se que os efeitos da pandemia persistiram mesmo após a fase crítica da crise sanitária, conforme dados de relatórios da OMS e da Organização das Nações Unidas^(42,43)^, evidenciando os impactos prolongados desse evento sobre a saúde física e mental dos profissionais.

À luz desses achados, os resultados deste estudo reforçam o Modelo Tripartido como referencial teórico para compreender o adoecimento mental por estresse, ansiedade e depressão na enfermagem. Ao destacar sintomas centrais que desempenham papel estratégico na rede, a análise evidencia como esses elementos se organizam e interagem entre si, ampliando a aplicabilidade do modelo ao contexto do trabalho em saúde.

### Limitações do estudo

Por não terem sido utilizados instrumentos específicos para avaliar o contexto organizacional, a análise de rede não considerou variáveis externas relevantes, como condições de trabalho, disponibilidade de recursos e suporte organizacional. Dessa forma, embora a rede tenha revelado a relação dinâmica entre sintomas de depressão, ansiedade e estresse, sua interpretação impossibilita a avaliação exata de condições organizacionais que podem estar relacionadas ao adoecimento mental de profissionais de enfermagem. Ademais, esse tipo de estudo impede a realização de análises de causalidade entre as variáveis estudadas.

### Contribuições para a área da enfermagem

A análise de rede da DASS-21 em profissionais de enfermagem traz contribuições importantes ao mostrar como os sintomas de depressão, ansiedade e estresse se conectam e se influenciam mutuamente. Essa abordagem permitiu identificar quais sintomas são mais relevantes para o inter relacionamento entre todos os nós e para a conectividade da rede como um todo, revelando um padrão de adoecimento próprio da categoria. Esses resultados podem direcionar estratégias de autocuidado dos profissionais e apoiar gestores de serviços de saúde no planejamento de ações mais direcionadas, focadas nos sintomas que mais impactam o bem-estar da equipe. Além disso, o estudo contribui para a área da enfermagem ao ampliar a base de evidências sustentadas por métodos robustos e ao oferecer uma nova forma de compreender o adoecimento mental relacionado ao trabalho.

## CONCLUSÕES

Os resultados deste estudo evidenciam que o adoecimento mental de trabalhadores de enfermagem se organiza em torno de sintomas centrais que desempenham papel fundamental na dinâmica da rede, influenciando a ativação e a manutenção de manifestações de depressão, ansiedade e estresse. A identificação desses sintomas centrais revela padrões estruturais que ajudam a compreender como o sofrimento emocional se articula na categoria, destacando pontos de vulnerabilidade que, quando não reconhecidos, podem amplificar os danos psicossociais do trabalho.

Esses achados fortalecem o potencial da DASS-21 como instrumento para o rastreamento de sintomas e oferecem subsídios consistentes para gestores, pesquisadores e formuladores de políticas na elaboração de estratégias de prevenção e gerenciamento de riscos psicossociais relacionados ao trabalho. Ao indicar quais sintomas exercem maior influência na rede, a análise orienta intervenções mais direcionadas e efetivas. De forma abrangente, os resultados reforçam a necessidade de políticas institucionais que promovam ambientes de trabalho psicologicamente seguros. Isso envolve práticas de monitoramento contínuo e apoio psicossocial. Além disso, é preciso reorganizar as condições laborais a fim de contribuir para a redução do sofrimento e promoção da saúde mental dos trabalhadores de enfermagem.

## Data Availability

Os dados de pesquisa só estão disponíveis mediante solicitação.
